# Lighting Deviation Correction for Integrating-Sphere Multispectral Imaging Systems

**DOI:** 10.3390/s19163501

**Published:** 2019-08-10

**Authors:** Zhe Zou, Hui-Liang Shen, Shijian Li, Yunfang Zhu, John H. Xin

**Affiliations:** 1 College of Information Science and Electronic Engineering, Zhejiang University, Hangzhou 310027, China; 2 College of Computer Science, Zhejiang University, Hangzhou 310027, China; 3 College of Computer Science and Information Engineering, Zhejiang Gongshang University, Hangzhou 310018, China; 4 Institute of Textiles and Clothing, The Hong Kong Polytechnic University, Hong Kong 999077, China

**Keywords:** multispectral imaging, integrating sphere, lighting deviation, spectral reflectance

## Abstract

In an integrating sphere multispectral imaging system, measurement inconsistency can arise when acquiring the spectral reflectances of samples. This is because the lighting condition can be changed by the measured samples, due to the multiple light reflections inside the integrating sphere. Besides, owing to non-uniform light transmission of the lens and narrow-band filters, the measured reflectance is spatially dependent. To deal with these problems, we propose a correction method that consists of two stages. The first stage employs a white board to correct non-uniformity and a small white patch to correct lighting deviation, both under the assumption of ideal Lambertian reflection. The second stage uses a polynomial regression model to further remove the lighting inconsistency when measuring non-Lambertian samples. The method is evaluated on image data acquired in a real multispectral imaging system. Experimental results illustrate that our method eliminates the measurement inconsistency considerably. This consequently improves the spectral and colorimetric accuracy in color measurement, which is crucial to practical applications.

## 1. Introduction

Integrating spheres are widely used in radiometric and photometric measurements, among which reflectance measurement is a typical application [1]. By collecting and integrating the reflected radiant flux, the aim of the sphere is to provide a stable and uniform illumination condition. The hemispherical reflectance or reflectance factor of a sample can then be measured in certain geometries.

Absolute or relative reflectance measurements can be conducted by using integrating spheres. In absolute measurements [2,3], the reflectance of a sample is measured by illuminating the sphere wall and the sample in turn. The relative measurements [4,5] introduce a reference standard of known reflectance; the reflectance of a sample is computed based on the ratio of detected signals corresponding to the sample and standard. There are two types of relative measurement methods. One is the comparison method, in which the standard and sample are placed in the sphere simultaneously. The other measures the standard and sample in the sphere successively and is known as the substitution method.

The spectrophotometer is a typical measurement system that uses the integrating sphere. It measures the average spectral reflectance from the sample port whose diameter is relatively large. Accordingly, it has a very coarse spatial resolution. A multispectral imaging system, when appropriately calibrated, can measure the spectral reflectance of a sample with spatially-fine resolution [6,7,8,9]. Currently, some multispectral imaging systems [10,11,12,13,14] use the integrating sphere to obtain a stable and uniform illumination. A monochrome camera, together with narrow-band filters or a liquid crystal tunable filter (LCTF) works as the detector. The detector port is usually on the opposite side of the sample port.

In a single-beam integrating sphere, substituting the standard with a sample will alter the throughput of the sphere, especially when the sample is of much lower reflectance than the standard. This usually leads to a lower measurement quantity. The measurement error caused by lighting deviation is known as the single-beam substitution error. Typical solutions to this problem include increasing the sphere size, decreasing the sample port area, using multiple standards with different reflectance levels and introducing an additional reference beam. The methods for eliminating substitution error have been investigated in [15,16].

In an integrating sphere multispectral imaging system, the sample port has a relatively large area and holds the standard and sample in turn, which introduces non-negligible lighting deviation. However, the solutions just mentioned focus on systems measuring the average spectral reflectance from the sample port and cannot be applied directly to the imaging system. Previous calibration methods [17,18,19,20] for the imaging system mainly dealt with the spatial non-uniformity, in which the dark current images and the images of the reference standard were usually involved. As these methods cannot eliminate lighting deviation, additional lighting correction methods are needed. The work [10] presented a Markov model to deal with this problem for the integrating sphere imaging system. The model is based on the assumption of constant lamp irradiance and Lambertian sample reflection. Considering the illuminant fluctuation and non-Lambertian samples, in this paper, we propose a two-stage lighting deviation correction for integrating sphere multispectral imaging systems.

The novelty of the proposed lighting correction is mainly two-fold. First, a small reference white patch is employed to compensate for the lighting deviation based on the assumption of Lambertian reflection. Second, a polynomial regression model is used to further reduce the measurement inconsistency of non-Lambertian samples in practical measurement. The improvement of measurement consistency is finally validated using intensive experiments.

## 2. The Problem of Lighting Deviation Correction

**Figure 1:**
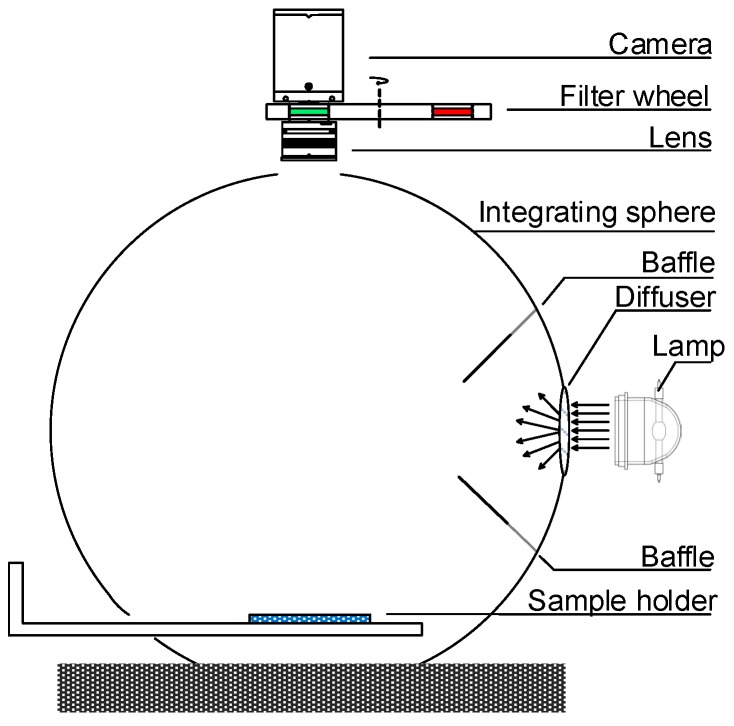
The schematic drawing of a typical integrating sphere multispectral imaging system.

Figure 1 illustrates a typical integrating sphere multispectral imaging system, which is of the d: 0${}^{{}^{\circ}}$ geometric condition. The system consists of an integrating sphere, a lamp, a monochrome camera, a lens, and a filter wheel installed with narrow-band filters. The incident light is diffused as it enters the sphere through the entrance port and is spatially integrated in the sphere. The multispectral image of a planar sample is acquired by rotating the filters into the optical path and acquiring band images through the detector port sequentially.

Lighting correction is needed for accurate and stable color measurement in an integrating sphere multispectral imaging system. The correction procedure mainly deals with two problems, i.e., spatial non-uniformity and lighting deviation. First, even if the integrating sphere provides a uniform illumination, the reflected fluxes reaching the image sensor from a uniform sample at different positions can still be different. This is due to the non-uniform light transmission of the lens and filters. This problem can be solved by acquiring an image of a white board that has the same size of the sample.

Second, lighting deviation can occur when measuring different samples. Due to the multiple light reflections inside the sphere, the sample itself can change the lighting condition; this is referred to as the background effect hereafter. This means that, for a certain small color patch, its measurements will be different if this patch is placed on different background samples. In addition to the imaged sample, the fluctuation of the lamp irradiance also leads to the lighting deviation.

To deal with the mentioned problems, we propose a two-stage lighting calibration method to correct the measurements of individual bands. In the first stage, we assume the sample surface is of ideal Lambertian diffuse reflection. We then characterize the spatial non-uniformity by acquiring the image of a white board, and correct lighting deviation by employing a small white patch for reference. In the second stage, we use a polynomial regression model to further reduce the background effect for practical color measurement.

The lighting deviation correction is performed on the camera response (i.e., the intensity of a pixel in the captured image). The spectral reflectance is reconstructed from the corrected camera responses using Wiener estimation [21]. To be more specific, in our 16-band multispectral imaging system, the 31-dimensional spectral reflectance for each pixel is mathematically reconstructed from the 16-dimensional camera response using a 31 × 16 reconstruction matrix. The reconstruction matrix is computed by acquiring the corrected camera responses of 144 color targets of known spectral reflectances and performing Wiener estimation. Note that the color targets and the measured sample are made of similar materials. Therefore, the consistency of the spectral reflectances can be improved by conducting the lighting deviation correction on the camera response.

## 3. Correction Stage I: White-Patch Normalization

**Figure 2:**
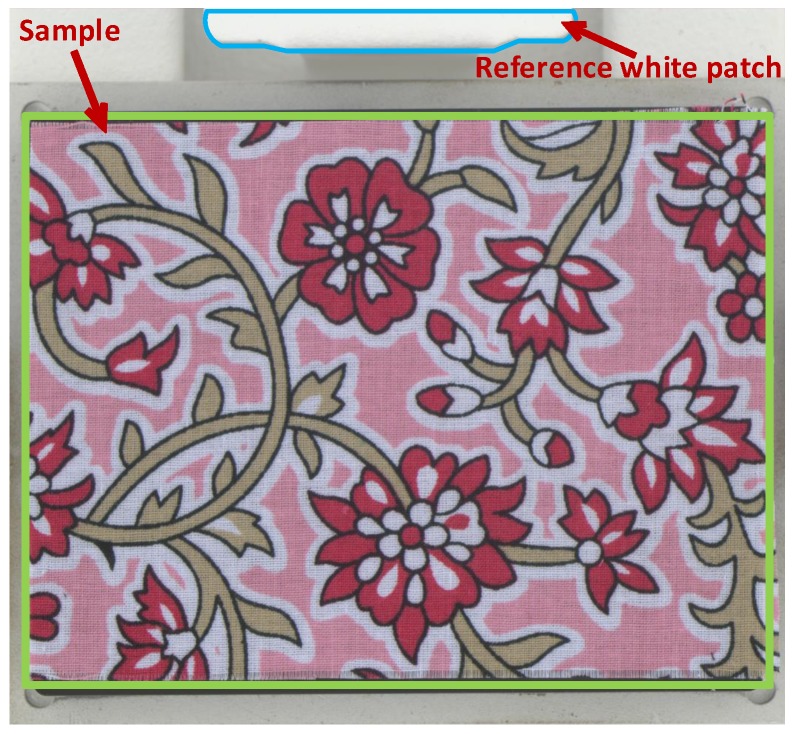
The layout of the small reference white patch and an imaged sample. The white patch is marked by a blue line, and the sample is marked by a green box.

Lambertian reflection is a common assumption in eliminating substitution errors for an integrating sphere [15,16]. In Stage I, we also employed this assumption in modeling the lighting deviation caused by the imaged samples. We used a white board, which is hereafter referred to as the white standard or simply the standard, to correct the spatial non-uniformity. We further employed a small white patch to model the change of lighting intensity caused by the imaged sample. Figure 2 shows the layout of the reference white patch and a sample. The size of the white patch was much smaller than that of the sample.

As illustrated in Figure 2, the lighting intensity inside the integrating sphere was recorded by a small reference white patch. It had similar properties as the sphere wall and was placed in the focal plane beside the sample holder. In the following, we introduce the theoretical analysis of non-uniformity correction and white-patch normalization for lighting calibration. In this stage, the white standard, white patch, and sample were all assumed to be of Lambertian reflection.

### 3.1. Using the White Standard to Correct Spatial Non-Uniformity

We denote the radiant flux entering the sphere in the wavelength range of λ±Δλ by $\Phi_{0}\left(\lambda \right)$, where Δλ is a quite small interval. Due to the existence of the diffuser and baffles, the flux can be assumed to strike the sphere wall uniformly. For the Lambertian sphere wall whose spectral reflectance is $\rho_{w}\left(\lambda \right)$, the flux in its first reflection is:

(1) $$\Phi_{1,w}\left(\lambda \right)=\rho_{w}\left(\lambda \right)\Phi_{0}\left(\lambda \right).$$

Among the flux reflected by the sphere wall, the percentages of the part striking the sphere wall and the part incident on the sample holder are denoted by $\alpha_{w,w}\in \left(0,1\right)$ and $\alpha_{w,\mathrm{sp}}\in \left(0,1\right)$, respectively. Due to the existence of the entrance port and detector port, we have $\alpha_{w,w}+\alpha_{w,\mathrm{sp}}<1$.

For calibration, the white standard was placed on the sample holder to acquire the spatial distribution of the lighting. The standard had the same size as the sample and was much larger than the white patch illustrated in Figure 2. The reflectance of the standard, ${\bar{\rho }}_{r}\left(\lambda \right)$, is also referred to as average reflectance. The flux in the first reflection of the standard under the integrating sphere lighting is computed as:

(2) $$\Phi_{1,r}\left(\lambda \right)=\alpha_{w,\mathrm{sp}}{\bar{\rho }}_{r}\left(\lambda \right)\Phi_{1,w}\left(\lambda \right).$$

A part of the flux reflected by the standard strikes the sphere wall, at a percentage of $\alpha_{\mathrm{sp},w}\in \left(0,\;1\right)$. The flux in the second reflection of the sphere wall can then be computed as:

(3) $$\Phi_{2,w}\left(\lambda \right)=\rho_{w}\left(\lambda \right)\cdot (\alpha_{w,w}\Phi_{1,w}\left(\lambda \right)+\alpha_{\mathrm{sp},w}\Phi_{1,r}\left(\lambda \right))=k_{r}\left(\lambda \right)\rho_{w}^{2}\left(\lambda \right)\Phi_{0}\left(\lambda \right),$$

where $k_{r}\left(\lambda \right):=\alpha_{w,w}+\alpha_{w,\mathrm{sp}}\alpha_{\mathrm{sp},w}{\bar{\rho }}_{r}\left(\lambda \right)$.

The flux in the second reflection of the standard is:

(4) $$\Phi_{2,r}\left(\lambda \right)=\alpha_{w,\mathrm{sp}}{\bar{\rho }}_{r}\left(\lambda \right)\Phi_{2,w}\left(\lambda \right).$$

Then, the flux in the sphere wall’s third reflection is computed as:

(5) $$\Phi_{3,w}\left(\lambda \right)=\rho_{w}\left(\lambda \right)\cdot (\alpha_{w,w}\Phi_{2,w}\left(\lambda \right)+\alpha_{\mathrm{sp},w}\Phi_{2,r}\left(\lambda \right))=k_{r}^{2}\left(\lambda \right)\rho_{w}^{3}\left(\lambda \right)\Phi_{0}\left(\lambda \right).$$

The flux in the $k^{\mathrm{th}}$ reflection of the sphere wall, $\Phi_{k,w}\left(\lambda \right)$, and that of the standard, $\Phi_{k,r}\left(\lambda \right)$, can be computed in a similar manner.

We first consider the imaging model of the white standard. For a pixel position $x={\left(x,y\right)}^{T}$ in the standard, we denote its spectral reflectance by $\rho_{r}\left(x,\lambda \right)$. A part of the flux reflected by the standard at x reaches the image sensor, at a percentage of β(x). Note that β(x) is spatially varying, due to the spatial non-uniformity as mentioned in Section 2. The intensity of the pixel x in the captured image, i.e., the camera response, is then computed as:

(6) $$\begin{array}{cc} u_{r}\left(x,\lambda \right) & =e\left(\lambda \right)\beta \left(x\right)\rho_{r}\left(x,\lambda \right)\cdot \frac{\alpha_{w,\mathrm{sp}}}{N}\sum_{k=1}^{\infty }\Phi_{k,w}\left(\lambda \right)\\ & =\rho_{r}\left(x,\lambda \right)\beta \left(x\right)\gamma \left(\lambda \right)\Phi_{0}\left(\lambda \right)\cdot \sum_{k=1}^{\infty }(k_{r}\left(\lambda \right)\rho_{w}\left(\lambda \right){)}^{k-1}, \end{array}$$

where e(λ) is the factor between the detected flux and camera response, *N* is the number of corresponding pixels of the standard, and $\gamma \left(\lambda \right):=\alpha_{w,\mathrm{sp}}\rho_{w}\left(\lambda \right)e\left(\lambda \right)/N$ is only determined by equipment specifications. Note that we subtract the dark current from the camera response in practical measurements. Since $0<\rho_{w}\left(\lambda \right)<1$ and $0<k_{r}\left(\lambda \right)<\alpha_{w,w}+\alpha_{w,\mathrm{sp}}<1$, we have $0<k_{r}\left(\lambda \right)\rho_{w}\left(\lambda \right)<1$. Equation (6) can be further computed as:

(7) $$u_{r}\left(x,\lambda \right)=\frac{\rho_{r}\left(x,\lambda \right)\beta \left(x\right)\gamma \left(\lambda \right)\Phi_{0}\left(\lambda \right)}{1-k_{r}\left(\lambda \right)\rho_{w}\left(\lambda \right)}.$$

Then, we consider the imaging model of a Lambertian sample whose average reflectance is ${\bar{\rho }}_{s}\left(\lambda \right)$. Assume that the radiant flux entering the sphere becomes $\Phi_{0}\left(\lambda \right)+\Delta \Phi_{0}\left(\lambda \right)$ due to the possible fluctuation of the lamp irradiance. The camera response at x becomes:

(8) $$u_{s}\left(x,\lambda \right)=\frac{\rho_{s}\left(x,\lambda \right)\beta \left(x\right)\gamma \left(\lambda \right)(\Phi_{0}\left(\lambda \right)+\Delta \Phi_{0}\left(\lambda \right))}{1-k_{s}\left(\lambda \right)\rho_{w}\left(\lambda \right)},$$

where $\rho_{s}\left(x,\lambda \right)$ is the sample reflectance at x, and $k_{s}\left(\lambda \right)$ is computed as:

(9) $$k_{s}\left(\lambda \right)=\alpha_{w,w}+\alpha_{w,\mathrm{sp}}\alpha_{\mathrm{sp},w}{\bar{\rho }}_{s}\left(\lambda \right).$$

The background variation, which corresponds to the different reflectances ${\bar{\rho }}_{s}\left(\lambda \right)$ of samples, leads to the change of $k_{s}\left(\lambda \right)$. Besides, the illuminant fluctuation introduces a time-variant $\Delta \Phi_{0}\left(\lambda \right)$. The lighting deviation in both cases influences the camera response $u_{s}\left(x,\lambda \right)$ of the sample, according to Equation (8).

The camera response of the sample $v_{s}\left(x,\lambda \right)$ can be normalized with respect to the response of the white standard at x,

(10) $$v_{s}\left(x,\lambda \right)=\frac{u_{s}\left(x,\lambda \right)}{u_{r}\left(x,\lambda \right)}=\frac{\rho_{s}\left(x,\lambda \right)}{\rho_{r}\left(x,\lambda \right)}\cdot q_{s}\left(\lambda \right),$$

where:

(11) $$q_{s}\left(\lambda \right)=\frac{1-k_{r}\left(\lambda \right)\rho_{w}\left(\lambda \right)}{1-k_{s}\left(\lambda \right)\rho_{w}\left(\lambda \right)}\cdot \frac{\Phi_{0}\left(\lambda \right)+\Delta \Phi_{0}\left(\lambda \right)}{\Phi_{0}\left(\lambda \right)}.$$

Note that $q_{s}\left(\lambda \right)$ varies with the lighting deviation just mentioned, due to the change of $k_{s}\left(\lambda \right)$ and $\Delta \Phi_{0}\left(\lambda \right)$ at the time of measurement.

Thanks to the uniformity of the white standard, we actually have $\rho_{r}\left(x,\lambda \right)={\bar{\rho }}_{r}\left(\lambda \right)$. By using the white standard, we eliminate the spatially-varying term β(x) from camera response $u_{s}\left(x,\lambda \right)$ and thus correct the spatial non-uniformity in Equation (10). However, the normalized camera response $v_{s}\left(x,\lambda \right)$ still suffers from the lighting deviation, due to the existence of term $q_{s}\left(\lambda \right)$.

### 3.2. Using the White Patch to Correct Lighting Deviation

We correct the lighting deviation using a reference white patch as illustrated in Figure 2. Thanks to the relatively large field of view of the imaging system, the camera responses of both the sample and reference white patch can be measured simultaneously. Let $\rho_{p}\left(x_{p},\lambda \right)$ be the spectral reflectance of the white patch at pixel $x_{p}$, its camera response when acquiring the image of the standard is:

(12) $$u_{r}\left(x_{p},\lambda \right)=\frac{\rho_{p}\left(x_{p},\lambda \right)\beta \left(x_{p}\right)\gamma \left(\lambda \right)\Phi_{0}\left(\lambda \right)}{1-k_{r}\left(\lambda \right)\rho_{w}\left(\lambda \right)},$$

and its camera response when acquiring the image of the sample is:

(13) $$u_{s}\left(x_{p},\lambda \right)=\frac{\rho_{p}\left(x_{p},\lambda \right)\beta \left(x_{p}\right)\gamma \left(\lambda \right)(\Phi_{0}\left(\lambda \right)+\Delta \Phi_{0}\left(\lambda \right))}{1-k_{s}\left(\lambda \right)\rho_{w}\left(\lambda \right)}.$$

Our aim is to characterize lighting deviation when imaging various samples; thus, we normalize $u_{s}\left(x_{p},\lambda \right)$ with respect to $u_{r}\left(x_{p},\lambda \right)$, yielding:

(14) $$v_{s}\left(x_{p},\lambda \right)=u_{s}\left(x_{p},\lambda \right)/u_{r}\left(x_{p},\lambda \right)=q_{s}\left(\lambda \right),$$

which has exactly the same form of $q_{s}\left(\lambda \right)$. We introduce a white-patch ratio defined as:

(15) $$p_{s}\left(\lambda \right):=1/v_{s}\left(x_{p},\lambda \right).$$

Then, the normalized camera response of the sample in Equation (10) can be further normalized, yielding:

(16) $${\overset{˘}{v}}_{s}\left(x,\lambda \right):=p_{s}\left(\lambda \right)v_{s}\left(x,\lambda \right)=\rho_{s}\left(x,\lambda \right)/\rho_{r}\left(x,\lambda \right).$$

When compared with Equation (10), it is observed that in Equation (16), $q_{s}\left(\lambda \right)$ is completely eliminated. We note that the white patch ratio actually characterizes the lighting change in the integrating sphere and can be regarded as another form of introducing a reference beam. Hence, lighting deviation can be corrected by employing a white patch for reference.

Based on the above theoretical analysis, we present the procedure of lighting correction using the white standard and white patch as follows. We first acquired the image of the white standard, obtaining the camera response $u_{r}\left(x,\lambda \right)$ of the white standard at position x and the camera response $u_{r}\left(x_{p},\lambda \right)$ of the reference white patch at position $x_{p}$. Then, we acquired the image of a sample, getting the camera responses of the sample and white patch, which are respectively denoted as $u_{s}\left(x,\lambda \right)$ and $u_{s}\left(x_{p},\lambda \right)$. Based on the white patch ratio $p_{s}\left(\lambda \right)=u_{r}\left(x_{p},\lambda \right)/u_{s}\left(x_{p},\lambda \right)$, the camera response after the correction is computed as:

(17) $${\overset{˘}{v}}_{s}\left(x,\lambda \right)=\frac{p_{s}\left(\lambda \right)u_{s}\left(x,\lambda \right)}{u_{r}\left(x,\lambda \right)}.$$

## 4. Correction Stage II: Polynomial Regression Modeling

We note that the lighting deviation cannot be fully corrected by using a white patch. This is due to the non-Lambertian nature of real surface reflection of the imaged sample. In [22,23], the deviation in reflectance measurements of non-Lambertian samples was observed. The integrating sphere multispectral imaging system can measure reflectance at the spatial resolution of camera pixels, but will suffer more from the variation of reflection characteristics in a sample. The influence of the material and texture structure on reflectance measurement was also investigated in previous works [24,25].

Owing to the bidirectional reflectance distribution function (BRDF) [26] of a non-Lambertian surface, the distribution of the reflected light varied with the incident angle. Due to the measurement geometry, the camera response exhibited an angular dependence. Therefore, among the reflected flux from the sample at position x, the percentage of the part reaching the image sensor changed in different reflections, rather than being a constant value of β(x) for a given x as assumed in Stage I. The imperfections of the sphere such as lack of symmetry also led to the deviation. Besides, the inter-reflections in a surface with texture structures may not be ignored. In addition, the stray light effect [27,28,29] also plays a role when a dark area is surrounded by bright regions or vice versa. As a result, the employment of a white patch in Stage I cannot totally remove the background effect. Actually, when samples are made of different materials, the problem becomes even more complex and an additional cross-media calibration is generally needed [24]. In the current work, we used textile samples made of similar materials for investigation.

The lighting deviation correction aims to produce similar camera responses for a given color regardless of lighting change. Since the influence of the aforementioned factors is rather complicated, it is difficult to introduce a complete physical model to correct lighting deviation. We instead resorted to polynomial regression [30,31], which is a machine learning approach, to further improve the consistency of camera responses. Our objective was that, when a given small patch is placed on different background samples, the corrected camera responses of the patch should be in good agreement with each other. The polynomial models were applied on individual multispectral bands.

In the polynomial model, the normalized camera response $v_{s}\left(x,\lambda \right)$ should be an input variables since it is the one to be corrected. The white-patch ratio $p_{s}\left(\lambda \right)$ should also be involved since it indicates the lighting change in the integrating sphere. For a given small patch placed on different background samples, a brighter background sample leads to a larger $v_{s}\left(x,\lambda \right)$ and hence a smaller $p_{s}\left(\lambda \right)$. Similarly, a smaller $v_{s}\left(x,\lambda \right)$ corresponds to a larger $p_{s}\left(\lambda \right)$. The polynomial model is thus able to produce close camera responses when the background sample changes. We found that a simple second-order polynomial model suffices for background effect removal,

(18) $${\tilde{v}}_{s}\left(x,\lambda \right)=c_{1}\left(\lambda \right)p_{s}\left(\lambda \right)v_{s}\left(x,\lambda \right)+c_{2}\left(\lambda \right)v_{s}\left(x,\lambda \right)+c_{3}\left(\lambda \right)p_{s}\left(\lambda \right)+c_{4}\left(\lambda \right),$$

where ${\tilde{v}}_{s}\left(x,\lambda \right)$ is the corrected camera response and $c_{k}\left(\lambda \right)$, 1≤k≤4, are the coefficients to be solved. The polynomial model can be of high orders or include additional terms, but our investigation indicated that this simple form sufficed for correcting lighting deviation and performed well on test data. We note that, however, as the polynomial model is phenomenological, it probably cannot be generalized to samples with reflection characteristics that differ from the training samples.

**Figure 3:**
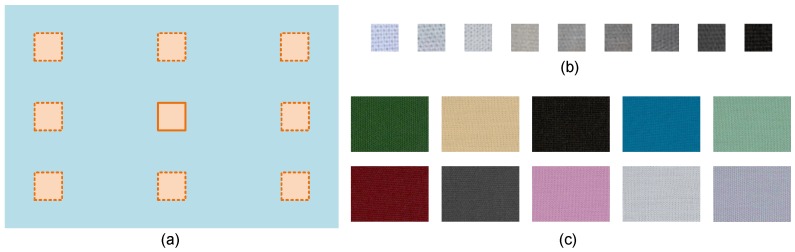
Training data collection. (**a**) Procedure of the measurement. A number of small patches are placed on various samples at different positions, respectively. The camera response of the small patch and the white-patch ratio are acquired in the imaging process. (**b**) Small gray patches of size 0.5 cm × 0.5 cm. The camera response of a small patch is averaged in a 35 × 35 pixel region. (**c**) Uniform background samples of size 10 cm × 8 cm.

We collected training data using the procedure illustrated in Figure 3a. We used a set of small gray patches (0.5 cm × 0.5 cm) as color targets and a set of color samples (10 cm × 8 cm) as backgrounds. The patches were placed on background samples at different positions. In total, $n_{p}=9$ small gray patches and $n_{b}=10$ background samples were used in the training procedure. The images of the small patches and background samples are shown in Figure 3b,c, respectively.

For the $i^{\mathrm{th}}$ patch and $j^{\mathrm{th}}$ background, we can get the normalized camera response $v_{s}^{i,j}\left(x,\lambda \right)$ of the patch and the white patch ratio $p_{s}^{i,j}\left(\lambda \right)$, as described in Section 3. Note that the normalized camera response was averaged in a 35 × 35 pixel region. To improve measurement consistency, the corrected responses of a patch placed on different backgrounds should be identical in the training data. Given a background whose index is $j_{\mathrm{ref}}$, the corrected response of the $i^{\mathrm{th}}$ patch in Stage I was ${\overset{˘}{v}}_{s}^{i,j_{\mathrm{ref}}}=v_{s}^{i,j_{\mathrm{ref}}}\left(x,\lambda \right)p_{s}^{i,j_{\mathrm{ref}}}\left(\lambda \right)$. For each *j*, we set the corrected response ${\tilde{v}}_{s}^{i,j}\left(x,\lambda \right)$, to the response on the $j_{\mathrm{ref}}^{\mathrm{th}}$ background, ${\overset{˘}{v}}_{s}^{i,j_{\mathrm{ref}}}$. In this way, a set of $n_{p}n_{b}$ training data was collected.

We concatenated the model coefficients into a vector $c={\left(c_{1}\left(\lambda \right),c_{2}\left(\lambda \right),c_{3}\left(\lambda \right),c_{4}\left(\lambda \right)\right)}^{T}$ and the training data into vectors as follows:

(19) $$\begin{array}{cc} v & ={\left(v_{1,1}\cdots v_{1,n_{b}}\;,\cdots \cdots ,v_{n_{p},1}\cdots v_{n_{p},n_{b}}\right)}^{T},\\ p & ={\left(p_{1,1}\cdots p_{1,n_{b}}\;,\cdots \cdots ,p_{n_{p},1}\cdots p_{n_{p},n_{b}}\right)}^{T},\\ \tilde{v} & ={\left({\tilde{v}}_{1,1}\cdots {\tilde{v}}_{1,n_{b}}\;,\cdots \cdots ,{\tilde{v}}_{n_{p},1}\cdots {\tilde{v}}_{n_{p},n_{b}}\right)}^{T}. \end{array}$$

Note that the variables x and λ are omitted in Equation (19) to simplify notation. We then construct a matrix M as:

(20) M=(p⊙v,v,p,1),

where ⊙ is the element-wise product of two vectors and $1\in R^{n_{p}n_{b}}$ is a vector of ones. According to Equation (18), the problem of estimating model coefficients can be formulated as:

(21) $$c^{*}=\arg\;\min_{c}\;{∥\tilde{v}-Mc∥}^{2},$$

which can be solved using least-squares as:

(22) $$c^{*}={\left(M^{T}M\right)}^{-1}M^{T}\tilde{v}.$$

After obtaining $c^{*}={\left(c_{1}^{*}\left(\lambda \right),c_{2}^{*}\left(\lambda \right),c_{3}^{*}\left(\lambda \right),c_{4}^{*}\left(\lambda \right)\right)}^{T}$, we can correct the camera response using the regression model in the measurement process. By acquiring images of the white standard and sample, we obtained the normalized camera response $v_{s}\left(x,\lambda \right)$ of the sample at position x and the white patch ratio $p_{s}\left(\lambda \right)$. The corrected camera response was then computed according to Equation (18), which gives:

(23) $${\tilde{v}}_{s}\left(x,\lambda \right)=(c_{1}^{*}\left(\lambda \right)p_{s}\left(\lambda \right)+c_{2}^{*}\left(\lambda \right))\cdot v_{s}\left(x,\lambda \right)+c_{3}^{*}\left(\lambda \right)p_{s}\left(\lambda \right)+c_{4}^{*}\left(\lambda \right).$$

## 5. Experimental Results

We built an integrating sphere multispectral imaging system whose structure was identical to that in Figure 1. The imaging system used a Hamamatsu 150 W super-quiet xenon lamp, a Zeiss 50 mm lens, a QImaging QI695 scientific monochrome camera, and a customized integrating sphere. The integrating sphere had a diameter of 50 cm. The size of the sample holder was 10 cm × 8 cm. The diameters of the entrance port and detector port were 6 cm and 5 cm, respectively. The port fraction of our integrating sphere was 1.63%, which was sufficient for good uniformity according to the sphere design rule [32]. The filter wheel was installed with 16 narrow-band filters. The full width at half maximum (FWHM) value of each filter was 10 nm, and the central wavelengths were at 400, 420, …, and 700 nm. By rotating the filter wheel, we recorded camera responses at different bands sequentially. The multispectral image of a sample was generated from normalized camera responses and corresponding white patch ratios. The 31-channel spectral reflectance of each pixel was reconstructed from the 16-band multispectral image using Wiener estimation [21].

Experiments were carried out to validate the proposed method on both training and test data acquired in our system. The improvement of measurement consistency was evaluated using the standard deviation (i.e., the square root of variance) of camera responses, as well as the spectral and colorimetric errors of spectral reflectances. Comparison with the state-of-the-art method was also performed on the test data.

### 5.1. Consistency Improvement on Camera Response

We first evaluated the improvement of response consistency using the training data shown in Figure 3. For a patch placed on various background samples, the consistency was computed as the standard deviation of its camera responses. The original camera responses were the normalized ones computed using Equation (10); camera responses in Stage I were computed using Equation (17); and those in Stage II were computed using Equation (23). The standard deviations of the training patches at each band are listed in Table 1. The standard deviations of the original data were relatively large in all bands. The standard deviation averaged on all bands was 0.0157 before lighting deviation correction. Its value reduced to 0.0067 in Stage I and further reduced to 0.0023 in Stage II. The final improvement of camera response consistency was 85.3%. For illustration, Table 2 lists the model coefficients obtained from the training data.

**Figure 4:**
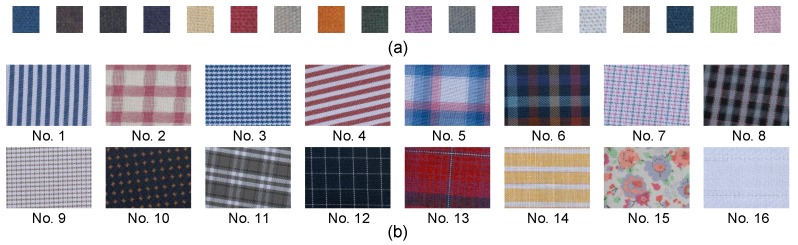
Test data collection. (**a**) Small color patches. (**b**) Background samples with various patterns.

We evaluated the model accuracy using the test data in Figure 4. As shown, the small patches were of different colors, and the background samples contained various color patterns. We note that these small patches and background samples were not used in polynomial regression modeling. As illustrated in Table 3, the original standard deviation averaged on all bands was 0.01. It dropped to 0.0051 in Stage I and further dropped to 0.0028 in Stage II. These results validate the generalization capability of the proposed lighting correction method.

**Figure 5:**
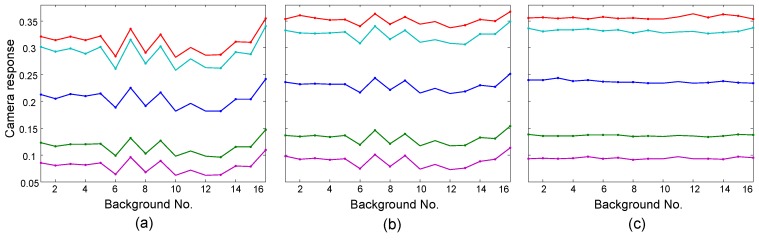
Camera responses of color patches with respect to backgrounds at the band of 440 nm. Curves in different colors correspond to different patches. (**a**) Original, (**b**) Stage I, and (**c**) Stage II.

Figure 5 presents the camera responses of some patches placed on different background samples, from the test data at Band No. 3 (440 nm). Each curve corresponds to a patch in the figure, and the fluctuation of the curve indicates the inconsistency. The fluctuations of the curves are quite evident in Figure 5a, while the fluctuations become smaller in Figure 5b. Figure 5c shows a great reduction in fluctuations, which indicates the removal of the background effect.

### 5.2. Consistency Improvement on Spectral Reflectance

By reconstructing the 31-channel spectral reflectance from corresponding camera responses at different bands, we further quantified the improvement in spectral color measurement with our method.

For a patch placed on different background samples, the consistency of color measurement can be evaluated using both spectral and colorimetric errors. The spectral error was computed as the root-mean-square (rms) between two spectral reflectances. The colorimetric errors were computed using the CIEDE2000 color difference formula [33] under CIE standard illuminants D65, A, and F2, respectively.

Table 4 lists the average spectral and colorimetric errors of the training data. The spectral rms error dropped from 0.1169 to 0.0556 in Stage I and finally dropped to 0.0149 in Stage II. The average color difference error under D65 of the original spectral reflectances was 5.1649 units before lighting deviation correction. It was reduced to 3.0092 and 0.3679 units after applying the corrections in Stage I and Stage II.

Table 5 shows the improvement of color measurement consistency on the test data. It is observed that both the spectral and colorimetric errors were considerably reduced in Stage I and Stage II. This was expected, as the consistency of camera responses were improved by our lighting correction method.

**Figure 6:**
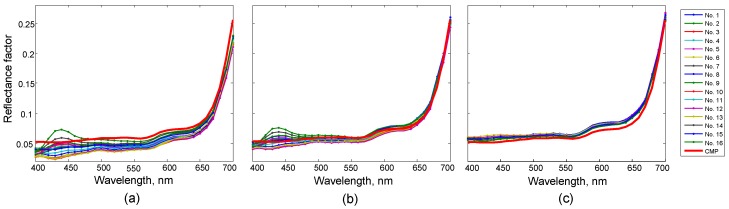
Spectral reflectance curves of a brown patch placed on different background samples. Note that the thick red curve corresponds to the spectral reflectance measured by a spectrophotometer. (**a**) Original reflectance curves. (**b**) Reflectance curves corrected in Stage I. (**c**) Reflectance curves corrected in Stage II.

For illustration, Figure 6 shows the spectral reflectance curves of a brown patch on different background samples. The curve of the spectral reflectance measured by a spectrophotometer is also presented for comparison. Note that due to the emission of fluorescent whitening agents in background samples, some of the curves in Figure 6a,b exhibited a conspicuous peak near 440 nm. The original reflectance curves were quite different due to the lighting deviation. The curves became closer to each other in Stage I and were in good agreement in Stage II.

### 5.3. Comparison with Existing Method

The Markov model [10] is the most relevant method to ours among previous works. It models the integrating sphere multispectral imaging of a Lambertian sample that may not be uniform and measures the reflectance of the sample at each pixel. We compared it with our method by computing measurement consistency on both camera response and spectral reflectance. The average standard deviations of camera responses, as well as average spectral and colorimetric errors of reconstructed reflectances are presented in Table 6. It is obvious that our method produced much lower errors under all metrics when compared with the Markov model.

## 6. Conclusions

We have proposed a lighting correction method to eliminate the measurement inconsistency caused by spatial non-uniformity and lighting deviation in integrating sphere multispectral imaging systems. We first modeled the imaging process of Lambertian surfaces in the integrating sphere, which explained the effect of these on measurements. Based on the theoretical analysis, we eliminated the spatial non-uniformity by acquiring the image of a white board and compensated for the lighting deviation by employing a reference white patch. We further introduced a polynomial regression model to correct the measurement inconsistency of real non-Lambertian samples. Experimental results validated that our method could improve measurement consistency considerably on both camera response and spectral reflectance. It also performed better than the state-of-the-art method.

## Figures and Tables

**Table 1:** Standard deviations computed from all color patches and background samples in the training data. The standard deviations averaged on all bands are also listed.

**Band No.**	**1**	**2**	**3**	**4**	**5**	**6**	**7**	**8**	
Original	0.0155	0.0172	0.0194	0.0159	0.0156	0.0128	0.0150	0.0129	
Stage I	0.0097	0.0102	0.0118	0.0082	0.0075	0.0049	0.0067	0.0049	
Stage II	0.0024	0.0023	0.0022	0.0022	0.0021	0.0021	0.0021	0.0021	
**Band No.**	**9**	**10**	**11**	**12**	**13**	**14**	**15**	**16**	**Average**
Original	0.0127	0.0146	0.0152	0.0137	0.0153	0.0155	0.0185	0.0212	0.0157
Stage I	0.0047	0.0061	0.0061	0.0047	0.0057	0.0048	0.0054	0.0059	0.0067
Stage II	0.0021	0.0020	0.0021	0.0021	0.0022	0.0024	0.0029	0.0032	0.0023

**Table 2:** The coefficients of the polynomial model obtained from the training data.

**Band No.**	**1**	**2**	**3**	**4**	**5**	**6**	**7**	**8**
***c*_1_**	0.8966	0.8787	0.9083	0.9168	0.9240	0.9438	0.9155	0.9293
***c*_2_**	0.1158	0.1337	0.1000	0.0901	0.0824	0.0613	0.0934	0.0792
***c*_3_**	0.2062	0.2034	0.2169	0.1507	0.1354	0.0837	0.1243	0.0849
***c*_4_**	−0.2229	−0.2193	−0.2329	−0.1617	−0.1468	−0.0913	−0.1374	−0.0943
**Band No.**	**9**	**10**	**11**	**12**	**13**	**14**	**15**	**16**
***c*_1_**	0.9445	0.9123	0.9155	0.9249	0.9118	0.9309	0.9276	0.9452
***c*_2_**	0.0608	0.0979	0.0931	0.0837	0.0972	0.0751	0.0778	0.0562
***c*_3_**	0.0782	0.1105	0.1059	0.0793	0.1000	0.0803	0.0951	0.1040
***c*_4_**	−0.0868	−0.1228	−0.1172	−0.0878	−0.1097	−0.0868	−0.1009	−0.1085

**Table 3:** Standard deviations of response consistency obtained from the test data when applying the polynomial regression model computed from the training data.

**Band No.**	**1**	**2**	**3**	**4**	**5**	**6**	**7**	**8**	
Original	0.0060	0.0133	0.0172	0.0113	0.0101	0.0081	0.0092	0.0081	
Stage I	0.0046	0.0082	0.0106	0.0063	0.0055	0.0039	0.0049	0.0039	
Stage II	0.0024	0.0029	0.0036	0.0027	0.0025	0.0023	0.0024	0.0024	
**Band No.**	**9**	**10**	**11**	**12**	**13**	**14**	**15**	**16**	**Average**
Original	0.0082	0.0095	0.0099	0.0096	0.0106	0.0100	0.0098	0.0096	0.0100
Stage I	0.0039	0.0046	0.0045	0.0040	0.0043	0.0039	0.0041	0.0046	0.0051
Stage II	0.0025	0.0025	0.0028	0.0029	0.0029	0.0031	0.0035	0.0042	0.0028

**Table 4:** Spectral reflectance consistency of the training data. Average spectral rms errors and color difference errors are listed.

	Spectral rms	$\Delta E_{00}$ (D65)	$\Delta E_{00}$ (A)	$\Delta E_{00}$ (F2)
Original	0.1169	5.1649	4.6991	5.3026
Stage I	0.0556	3.0092	2.6734	3.1455
Stage II	0.0149	0.3679	0.3573	0.3634

**Table 5:** Spectral reflectance consistency of the test data. Average spectral rms errors and color difference errors are listed.

	Spectral rms	$\Delta E_{00}$ (D65)	$\Delta E_{00}$ (A)	$\Delta E_{00}$ (F2)
Original	0.1240	4.1274	3.9949	4.1644
Stage I	0.0617	1.7949	1.6909	1.8808
Stage II	0.0201	0.5821	0.5712	0.6020

**Table 6:** Comparison with the Markov model [10] on measurement consistency. Average standard deviations of camera responses, spectral rms errors, and color difference errors of the test data are listed.

	Response std. dev.	Spectral rms	$\Delta E_{00}$ (D65)	$\Delta E_{00}$ (A)	$\Delta E_{00}$ (F2)
Original	0.0100	0.1240	4.1274	3.9949	4.1644
Markov model	0.0044	0.0625	1.7368	1.6907	1.8580
Ours	0.0028	0.0201	0.5821	0.5712	0.6020
